# Gait mechanics contribute to exercise induced pain flares in knee osteoarthritis

**DOI:** 10.1186/s12891-019-2493-4

**Published:** 2019-03-14

**Authors:** Katherine A. Boyer, Jocelyn F. Hafer

**Affiliations:** 1 Department of Kinesiology, University of Massachusetts-Amherst, 110 Totman Building 30 Eastman Lane, Amherst, MA 01003 USA; 2 University of Massachusetts-Amherst, Mechanical and Industrial Engineering, Amherst, USA; 3 Department of Orthopedics and Physical Rehabilitation, University of Massachusetts Medical School, Worcester, USA; 4 School of Kinesiology, University of Michigan, Central Campus Recreation Building, 401 Washtenaw Avenue, Ann Arbor, MI 48109 USA

**Keywords:** Knee osteoarthritis, Gait mechanics, Exercise induced pain flare; muscle activation

## Abstract

**Background:**

Exercise-induced pain flares represent a significant barrier for individuals with knee osteoarthritis to meet physical activity recommendations. There is a need to understand factors that contribute to pain flares and the potential for the motor system to adapt and reduce joint loading should a flare occur. The study aim was to examine the impact of a bout of exercise on self-reported pain, walking mechanics and muscle co-contraction for participants with knee osteoarthritis.

**Methods:**

Thirty-six adults (17 healthy older and 19 knee osteoarthritis) participated in this study. Self-reported pain, joint mechanics and muscle co-activation during gait at two self-selected speeds were collected before and after a 20-min preferred pace treadmill walk (20MTW).

**Results:**

Eight of nineteen osteoarthritis participants had a clinically significant pain flare response to the 20MTW. At baseline the participants that did not experience a pain flare had smaller knee flexion and total reaction moments compared to both the participants with pain flares (*p* = 0.02; *p* = 0.05) and controls (*p* < 0.001; *p* < 0.001). In addition, the 2nd peak knee adduction (*p* = 0.01) and internal rotation (*p* = 0.001) moments were smaller in the no flares as compared to controls. The pain flare participants differed from controls with smaller knee internal rotation moments (*p* = 0.03), but greater relative hamstrings (vs. quadriceps) and medial (vs. lateral) muscle activation (*p* = 0.04, *p* = 0.04) compared to both controls and no flare participants (p = 0.04, *p* = 0.007). Following the 20MTW there were greater decreases in the 1st and 2nd peak knee adduction (*p* = 0.03; *p* = 0.02), and internal rotation (*p* = 0.002) moments for the pain flare as compared to the no flare group. In addition, for the pain flare as compared to controls, greater decreases in the knee flexion (*p* = 0.03) and internal rotation (*p* = 0.005) moments were found.

**Conclusions:**

Individuals who adapt their gait to reduce knee joint loads may be less susceptible to exercise-induced pain flares. This highlights a potential role of gait biomechanics in short-term osteoarthritis pain fluctuations. The results also suggest that despite the chronic nature of osteoarthritis pain, the motor system’s ability to respond to nociceptive stimuli remains intact.

**Electronic supplementary material:**

The online version of this article (10.1186/s12891-019-2493-4) contains supplementary material, which is available to authorized users.

## Background

Knee osteoarthritis (OA) is one of the most common sources of musculoskeletal pain in adults over the age of 50 [1]. Pain and symptom management are the primary targets for clinical care of knee OA as there are no widely available disease modifying treatments. While use of analgesic and anti-inflammatory drugs is common, the primary recommendation for long term symptom management is exercise [2]. However, single bouts of weight-bearing exercise are known to acutely exacerbate pain, making movement-evoked pain flares (i.e., acute increases in pain) a characteristic symptom of knee OA [3–5]. These pain flares and the associated decreases in physical performance [6] can contribute to poor quality of life for individuals with knee OA. Pain flares also represent a major challenge to patient compliance with exercise prescription and programs, which is problematic as adherence to exercise training reduces pain flare magnitude over time [2, 5]. As pain and variations in pain through-out a day remain a target for most OA treatments, determining the mechanisms at play in exercise induced pain is essential.

Gait biomechanics may play an important role in exercise induced pain flares. When compared to age-matched healthy adults, it’s well documented that OA patients exhibit altered gait mechanics [7–9]. Within and between person variations in gait mechanics can alter muscle, external and soft tissue forces in the knee and may change the mechanical stimuli in joint tissue that would contribute to pain [10]. Initial cross sectional studies suggest a relationship between the knee adduction moment, a surrogate measure of the distribution of load between the medial and lateral compartments of the knee, and OA symptomatic and radiographic severity [8, 11–13]. Further, in a within patient analysis, a positive association between knees with pain on walking and the knee adduction moment magnitude during a subsequent walk has been reported [14]. Finally, when peak knee moments are reduced using shoe interventions, clinically relevant reductions in chronic joint pain have been reported [15]. Together these studies suggest that variations in gait mechanics, in particular the external knee flexion and adduction moments, can alter the pain experienced in knee OA, however whether greater moments might contribute to pain flares during exercise is not clear.

One of the challenges in quantifying the role of variations in gait mechanics on pain in OA is that gait mechanics may act as both a stimulus for pain and/or be altered as part of a motor system response to joint pain. The leading theories of pain-related movement adaptations suggest several neuromuscular responses that would lead to biomechanical adaptations such as increases in stiffness and decreases in joint movement and a reduction or redistribution of the total load on the painful joint [16–18]. In healthy young adults, acute pain induced by injection of hypertonic saline causes decreases in knee moments and quadriceps activation that are similar to gait adaptations seen with OA pain [19–21]. These studies provide evidence of the nature of the biomechanical response to knee joint pain however, the application of these studies’ findings to knee OA may be limited because gait and pain response may differ between young adults and adults who are more similar in age to individuals with knee OA. Given the negative impact of intermittent pain on physical performance there is a need to understand if and how individuals modify their biomechanics in response to increases in knee joint pain. Elucidating this response is necessary to understand both the mechanisms and targets for management of changes in performance with intermittent pain.

Typical cross-sectional or longitudinal studies preclude an evaluation of nociception-motor interactions and gait compensations that may be attributed to acute pain, as opposed to longer-term factors such as structural changes, chronic pain or learned gait compensations. The acute increase in pain in response to a mechanical stimulus (i.e. weight bearing exercise) presents an opportunity to probe the relationship between gait mechanics and changes in pain in older adults in the absence of changes in disease severity. Prior work quantifying the efficacy of pain pharmacology used a treadmill walking bout to produce an acute pain flare [4, 22]. A similar protocol may be able to discern the contribution to or response of gait mechanics to acute exercise-induced pain flares. Therefore, the aims of this study were to quantify 1) the impact of baseline knee joint mechanics and co-activation on changes in OA pain severity in response to a bout of treadmill walking and 2) the biomechanical response to increased pain severity. It was hypothesized that larger knee joint moments and greater muscle co-activation would yield greater pain flares with walking. In addition we hypothesized that there would be increases in perceived pain along with a reduction in the knee flexion angles, peak knee joint moments and an increase in co-activation of muscles crossing the knee joint in response to a bout of treadmill walking.

## Methods

Participants with and without symptomatic knee OA were recruited from surrounding communities via flyer, advertisements and word of mouth. All participants provided written informed consent as approved by the University of Massachusetts-Amherst Internal Review Boards. A power analysis was completed using data from the literature on knee OA gait with pain [15, 23] and indicated group sizes of *n* = 6 to 12 were needed to detect 10–20% differences in knee kinematic and kinetics parameters with a power of β = 0.8 and corresponding effect sizes ranging from 0.4–1.5. Inclusion criteria were ages 50–75 years, BMI < 35 kg/m^2^, good general health, ability to walk unaided, and no history of cardiovascular or neurological disorders. Participants for the OA group met the American College of Rheumatology clinical classification criteria for OA in at least 1 knee and reported physician-diagnosed knee OA [24]. Prior to participation in study activities, participants first completed an IRB approved informed consent document and the Physical Activity Readiness Questionnaire for Everyone to assess risk factors for exercise participation. Participants were asked to refrain from taking pain medication for 24 h prior to their study visit. Knee OA symptom severity and physical function were captured using the Knee Osteoarthritis Outcome Score (KOOS) [25]. Participants then completed a standardized testing protocol that included reporting pain on a verbal numeric rating scale (vNRS), overground gait analysis, self-paced treadmill walk, and a repetition of the pain reporting and overground gait analysis.

### Overground gait analysis

Participants completed 3 walking trials at *preferred* pace and then at a *faster than preferred* pace over a 25 m walkway while kinematic and kinetic data were collected. For the *faster than preferred* condition, participants were instructed to walk as if they were “trying to catch a bus”. The Point Cluster Technique (PCT) marker set up was used on the OA participants’ more affected limb and the right limb for controls [26]. With the PCT marker protocol, clusters of nine and seven reflective markers are distributed on the thigh and shank, respectively. Cluster coordinate systems are determined for the thigh and shank separately by calculating principal axes of the clusters assuming a unit weight for each marker. During a static reference trial, markers placed on bilateral greater trochanter, posterior superior iliac spine, anterior superior iliac spine; medial and lateral femoral epicondyles, tibia plateau, and malleoli; 5th metatarsal head and heel; and the marker clusters establish the tibial, femoral, foot and pelvic anatomic coordinate systems. The relative position and orientation between the marker cluster coordinate systems and the anatomical coordinate systems are calculated in the reference trial. Joint angles are calculated as projected angles and joint moments are calculated via inverse dynamics and reported as external moments, resolved in the distal coordinate system. Due to problems with marker occlusion, gait data could not be used for 1 knee OA and 2 control participants.

### Exercise protocol

Participants completed a 20 min treadmill walk (20MTW) at preferred walking pace. Treadmill speed was started below participants’ overground preferred walking speed and then increased or decreased in increments of 0.1 mph until participants indicated that the pace felt normal and could be sustained for 20 min. Perceived pain was evaluated on an 11 point vNRS every two minutes throughout the treadmill walk. The pain ratings in the first and final 2 min of the 20MTW were used to evaluate acute changes in pain in response to exercise.

### Directed co-contraction ratio

Electromyography (EMG, Trigno Delsys, MA, USA) was collected at 2000 Hz during the second and last minutes of the 20MTW. Electrodes were placed over the rectus femoris, vastus lateralis, vastus medialis, biceps femoris, semitendinosus, medial and lateral gastrocnemii and tibialis anterior according to SENIAM guidelines [27]. Raw EMG data had offset removed, were band-pass filtered (20–500 Hz), full wave rectified and then filtered with a zero lag, fourth order, 20 Hz low pass Butterworth filter to create linear envelopes using custom MatLab code. Heelstrike and toe-off were identified using an accelerometer placed on the lower leg. EMG for each muscle was then normalized to the average stance phase activity from 10 strides in the second minute of the 20MTW [28]. Directed co-contraction ratios (DCCR) were calculated to compare relative activation between the knee extensors (rectus femoris and vasti) and knee flexors (hamstrings and gastrocnemii) as well as between lateral (vastus lateralis, biceps femoris, and lateral gastrocnemius) and medial (vastus medialis, semitendinosus, and medial gastrocnemius) knee muscles [29]. The DCCR was calculated at each data point *t* for each stride *s* using one of two equations:

For the extensors vs. flexors ratio, if extensor activation was greater than flexor activation:

$$ {DCCR}_{t,s}=1-\frac{{\left( average\ of\ flexor\ linear\ envelopes\right)}_{t,s}}{{\left( average\ of\ extensor\ linear\ envelopes\right)}_{t,s}} $$

Else

$$ {DCCR}_{t,s}=\frac{{\left( average\ of\ extensor\ linear\ envelopes\right)}_{t,s}}{{\left( average\ of\ flexor\ linear\ envelopes\right)}_{t,s}}-1 $$

The same procedure was followed for the lateral vs. medial ratio with lateral muscles replacing extensors and medial muscles replacing flexors in the above equations. For DCCRs, values closer to 1 or − 1 indicate activation that is primarily due to one group in the ratio (for + 1, greater extensor or lateral activation; for − 1, greater flexor or medial activation). Values close to 0 indicate relatively equal activation of both muscle groups in the ratio. DCCRs for the extensor:flexor and lateral:medial comparisons were averaged over terminal swing (last 15% of swing) and early, mid, and late (thirds of) stance. Due to technical issues during the data collection EMG data for 4 OA and 3 controls were excluded from the analysis.

### Primary outcomes

The primary outcomes for this study were selected based on proposed pain-induced motor system adaptation strategies [16–18]. These proposed pain adaptations include: an increase in stiffness and decrease in joint movement (knee flexion angles at foot contact, loading response peak and toe-off, and increased co-activation of muscles crossing the knee); a reduction in total load on the painful joint (vertical ground reaction force, knee flexion and internal rotation and total reaction moment); and a redistribution of load across or within the medial and lateral compartments (1st and 2nd peak knee adduction moments, mean knee internal-external rotation angle over stance). The total reaction moment was calculated as the root-mean-square of the three components of the knee joint moments [30]. The total reaction moment is a resultant measure and a surrogate measure of the total load on the medial compartment. Secondary outcome measures to gain insight to compensatory strategies in OA and with pain included: ankle and hip flexion angles at heel-strike, ankle and hip range of motion in stance, peak ankle eversion angle, peak hip flexion, extension and 1st peak adduction moment and peak ankle plantar flexion, dorsiflexion and eversion moments.

### Statistical analysis

Preliminary evaluation of the pain changes in response to the 20MTW indicated that not all participants experienced a clinically important difference in pain (i.e., flare). Thus, OA participants were split into pain flare and no flare groups based on changes in vNRS. Participants who reported a change in pain ≥1 point in response to the 20MTW were assigned to the pain flare group. A ≥ 1 point change is considered a minimal clinically important difference on the vNRS for individuals with mild to moderate baseline pain [31, 32]. Un-paired student’s t-tests were used to test for differences in participant characteristics and patient reported outcomes between pain flare and no flare OA groups. Two-way ANOVAs (α = 0.05) were used to test for significant group, condition (preferred or faster than preferred) and group by condition interaction effects at baseline and for changes in the overground kinematics and kinetics in response to the 20MTW. One-way ANOVAs were used to test for an effect of group for co-activation during the 2nd minute of treadmill walking and the change between the 2nd and 20th minute of treadmill walking. Least significant difference post-hoc analysis was used to quantify pair-wise group differences where main effects were found. Cohen’s d effect sizes of the differences were calculated and a medium effect size was considered d > 0.5 and a large effect d > 0.8. Of note, an enrollment target of 18 for the OA group was made to power the study to test the hypothesis that the change in pain was significantly different from zero using literature data [4]. However, the pain response of our participants was very different from the prior study and as such, the study is not powered to test for differences in the change in pain with these subgroupings.

## Results

Thirty-six adults (17 healthy older and 19 with mild to moderate symptomatic knee OA) were enrolled in this study. Eight of nineteen OA participants had a significant flare response to the 20MTW. There were no OA group differences in the treadmill speed for the 20MTW (*p* = 0.89), demographics (age, *p* = 0.85 and BMI, *p* = 0.9) or participant reported OA symptoms (KOOS pain, *p* = 0.09 and ADL function, *p* = 0.16) (Table 1).

**Fig. 1:**
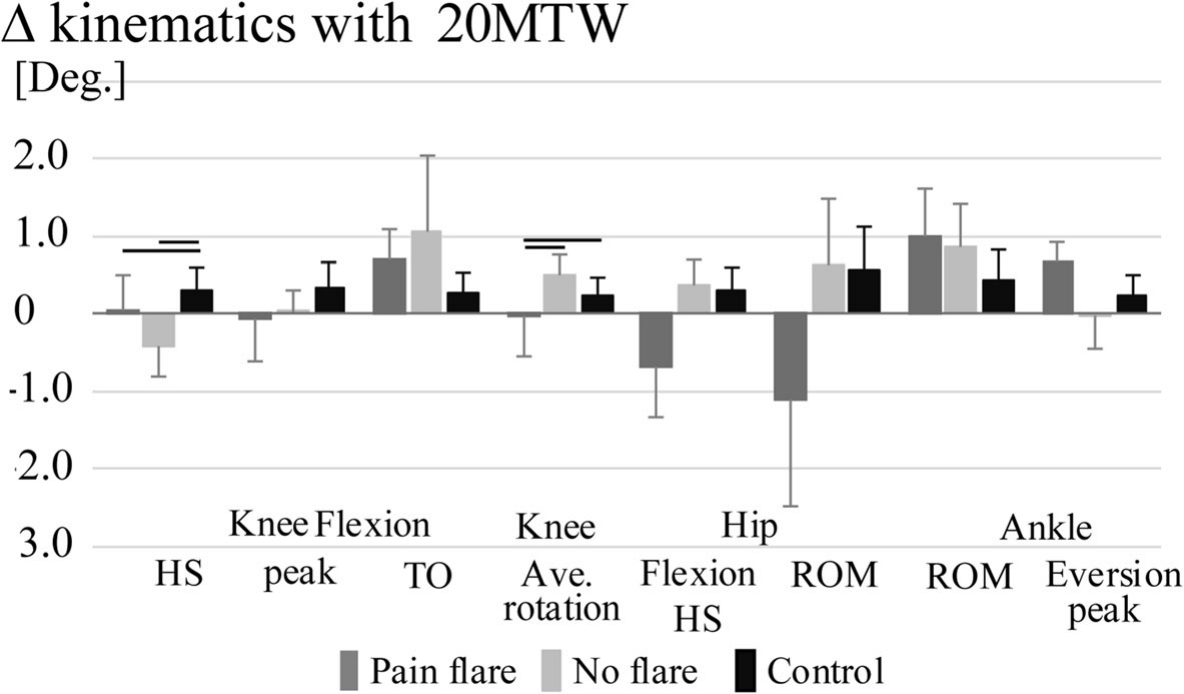
Mean changes (±SE) in hip, knee and ankle kinematics with the 20-min treadmill walk (20MTW). Black bars indicate significant post-hoc group differences α = 0.05

**Table 1:** Group demographics (Mean and SE) including preferred treadmill walking speed and participant reported symptoms at baseline and the change with the 20MTW. vNRS: verbal numerical rating scale pain measure

Normal speed	Age	BMI (kg/m^2^)	Treadmill Speed (m/s)	Baseline vNRS	Change in pain (vNRS)	KOOS Pain	KOOS ADL
Pain Flare (*n* = 8; 3F)	62.1 (1.9)	25.89 (1.47)	0.92 (0.13)	2.63 (1.00)	1.50 (0.27)^a^	59.1 (7.09)	68.1 (8.5)
No Flare (*n* = 11;10F)	62.6 (1.9)	25.69 (1.01)	0.90 (0.07)	1.36 (0.53)	−0.05 (0.11)^a^	70.7 (4.19)	79.3 (3.0)
Controls (*n* = 17;14F)	64.5 (1.5)	24.3 (0.9)	1.08 (0.06)	–	–	–	–

^a^indicates a significant difference between OA groups

### Baseline

At baseline, group effects were found for overground walking speed (*p* = 0.04), cadence (*p* = 0.05) heel-strike and toe off knee flexion angles (*p* < 0.001 for both), knee internal-external rotation angle over stance (*p* = 0.05), and heel-strike hip flexion angle (*p* = 0.001) (Table 2 and Additional file 1: Table S1). There were no group by condition interaction effects. Post-hoc comparisons indicated that OA groups walked slower and did not achieve as much knee extension (at heel-strike) or flexion (at toe-off) as healthy controls. In addition, the pain flare group walked with a slower cadence, and a more internally rotated femur relative to the tibia compared to both the no flare and controls.

**Table 2:** Baseline knee angles for preferred speed overground walking, Mean (SE)

	Overground speed^a^ (m/s)	Cadence^a^ (step/min)	Heel-strike Flexion^a^ (deg)	Peak Flexion (deg)	Toe-off Flexion^a^ (deg)	Average In/ext. rotation^a^ (deg)
Pain Flare (*n* = 8)	1.35 (0.10)	58.28 (1.51)	9.71 (2.76)	20.49 (2.44)	58.25 (3.50)	3.07 (1.20)
No Flare (*n* = 11)	1.35 (0.06)	59.41 (0.90)	6.70 (1.31)	16.06 (1.68)	62.33 (1.97)	−1.47 (1.63)
Controls (*n* = 15)	1.48 (0.03)	60.43 (0.97)	1.66 (1.14)	18.64 (1.15)	67.36 (0.91)	−0.04 (1.25)
*p*-value /effect size						
PF vs C	*p* = 0.04 d = 0.83	*p* = 0.02 d = 0.78	*p* < 0.001 d = 1.48		*p* < 0.001 d = 1.51	*p* = 0.05 d = 0.70
NF vs C	*p* = 0.09 d = 0.74		*p* = 0.002 d = 1.00		*p* = 0.002 d = 0.98	
PF vs NF		*p* = 0.05 d = 0.60				*p* = 0.02 d = 0.95

^a^indicates a significant main group effect. The final rows report the *p*-values and cohen’s d effect size for post-hoc pairwise comparisons with *p* < 0.1 and d > 0.4

Knee flexion (*p* < 0.001), 2nd peak knee adduction (*p* = 0.04), knee internal rotation (*p* = 0.002), total knee reaction (*p* = 0.001), hip extension (p < 0.001), 1st peak hip adduction (p = 0.001), and peak ankle plantar-flexion moments (*p* = 0.003) differed between groups (Tables 3 and 4 and Additional file 2: Table S2). At baseline the no flare group had smaller knee flexion and total reaction moments compared to both the pain flare and control groups. In comparison to the pain flare group only, the no flare group had smaller peak ankle plantarflexion moments and displayed a medium effect size for a smaller in 2nd peak knee adduction moment. In comparison to the control group only, the no flare group had smaller 2nd peak knee adduction, knee internal rotation moments as well as smaller hip flexion, extension and 1st peak adduction moments. Only the knee internal rotation, hip extension and 1st peak hip adduction moments were smaller for the pain flare as compared to control group. There was a group effect for muscle co-activation (DCCR) in late stance for both muscle group comparisons (Table 5). The flare group displayed greater relative hamstrings (vs. quadriceps) and medial (vs. lateral) activation compared to the controls (*p* = 0.04, *p* = 0.04) and the no flare groups (*p* = 0.04, *p* = 0.007).

**Table 3:** Baseline external knee moments (%BW x Ht) for preferred speed overground walking, Mean (SE)

	1st Peak vertical GRF BW	Flexion^a^	1st Adduction	2nd Adduction^a^	Internal rotation^a^	Total reaction^a^
Pain Flare (*n* = 7)	1.16 (0.03)	3.65 (0.58)	−2.94 (0.34)	−1.95 (0.32)	−0.76 (0.13)	4.85 (0.46)
No Flare (*n* = 11)	1.14 (0.04)	2.30 (0.39)	−2.73 (0.29)	−1.44 (0.24)	−0.73 (0.09)	3.73 (0.34)
Controls (*n* = 15)	1.22 (0.02)	4.10 (0.31)	−3.33 (0.33)	−2.04 (0.31)	−1.03 (0.09)	5.38 (0.37)
*p*-value/effect size						
PF vs C					*p* = 0.03 d = 0.66	
NF vs C		*p* < 0.001, d = 1.24		*p* = 0.01, d = 0.67	*p* = 0.001 d = 1.00	*p* < 0.001, d = 1.12
PF vs NF		*p* = 0.02 d = 0.84		*p* = 0.1 d = 0.7		*p* = 0.05 d = 0.84

^a^indicates a significant group effect. The final rows report the *p*-values and cohen’s d effect size for post-hoc pairwise comparisons with *p* < 0.1 and d > 0.4

**Table 4:** Baseline values for the hip and ankle outcome measures at the preferred walking pace. The last row reports the *p*-values and cohen’s d effect size for post-hoc pairwise comparisons with *p* < 0.1 and d > 0.4

	Hip Angle (deg)		Ankle Angles (deg)		Hip Moments (% BW * Ht)			Ankle Moments (% BW * Ht)		
	Heel-strike flexion	Flexion ROM	Flexion ROM	Peak Eversion	Flexion	Extension	1st Adduction	PlantarFlexion	Dorsi-Flexion	Eversion
Pain Flare	43.70	42.06	26.19	2.56	−4.40	3.84	−5.52	1.75	−7.99	0.84
SE	1.77	3.10	2.29	1.33	0.70	0.37	0.53	0.13	0.61	0.29
No Flare	38.28	45.78	28.78	6.05	−3.20	4.10	−5.73	1.22	−8.58	0.58
SE	2.00	2.16	2.24	1.09	0.37	0.22	0.31	0.11	0.26	0.10
Control	34.51	47.37	29.91	4.03	−4.53	5.41	−6.74	1.91	−8.90	0.55
SE	2.01	1.11	1.16	0.76	0.27	0.31	0.21	0.18	0.26	0.08
*p*-value/effect size										
PF Vs C						*p* < 0.001, d = 1.10	*p* < 0.001, d = 1.08			
NP vs C					*p* < 0.001 d = 0.77	*p* < 0.001, d = 0.90	*p* < 0.001, d = 1.02			
PF vs NF								*p* = 0.04 d = 1.40

**Table 5:** Baseline and change in DCCR for the quadriceps: hamstrings and medial: lateral muscle grouping

		Quadriceps: Hamstrings				Medial: Lateral			
		Terminal Swing	Early Stance	Mid stance	Late Stance	Terminal Swing	Early Stance	Mid stance	Late Stance
Baseline	Pain flare	−0.37	0.12	−0.04	−0.26*	−0.02	0.01	0.06	−0.15*
	SE	0.1	0.04	0.09	0.08	0.11	0.03	0.06	0.07
	No flare	−0.29	0.09	0.00	0.04*	−0.04	− 0.03	− 0.02	0.1*
	SE	0.09	0.03	0.04	0.09	0.07	0.03	0.05	0.04
	Controls	−0.42	0.04	0.04	0.02*	0.01	0.01	−0.04	0.03*
	SE	0.04	0.03	0.05	0.06	0.07	0.02	0.04	0.04
Change with 20MTW	Pain flare	0.00	−0.12	0.19	0.11	− 0.08	−0.08	0.09	0.13
	SE	0.07	0.09	0.08	0.09	0.06	0.06	0.06	0.06
	No flare	0.07	−0.06	−0.07	−0.03	−0.01	− 0.01	0.13	0.07
	SE	0.11	0.08	0.05	0.07	0.07	0.07	0.09	0.06
	Controls	0.13	0.06	0.12	0.13	−0.04	−0.04	−0.04	−0.01
	SE	0.08	0.06	0.09	0.11	0.05	0.05	0.04	0.06

* indicates *p* < 0.05 for the pain compared to other groups

### Response to 20MTW

In response to the 20MTW there was a group effect for the change in heel-strike knee flexion angle (*p* = 0.04) and mean knee internal-external rotation angle over stance (*p* = 0.05) (Fig. 1). There was also a condition effect indicating a greater magnitude of change with the 20MTW in the faster than preferred condition for walking speed (*p* = 0.002), cadence (*p* = 0.02), vertical ground reaction force (*p* = 0.05) and hip flexion range of motion (*p* = 0.002). There were no group by condition interaction effects. There was a difference in the change in knee flexion at heel-strike for the pain flare (*p* = 0.05, d = 0.52) and no flare (*p* = 0.03, d = 0.28) as compared to the controls. In addition, the change in average internal tibia rotation with respect to the femur was greater for the pain flare as compared to both no flare and controls (*p* = 0.02, d = 0.66 & *p* = 0.04, d = 0.41 respectively). However, the average change in kinematics was less than 1 degree for all outcomes.

**Fig. 2:**
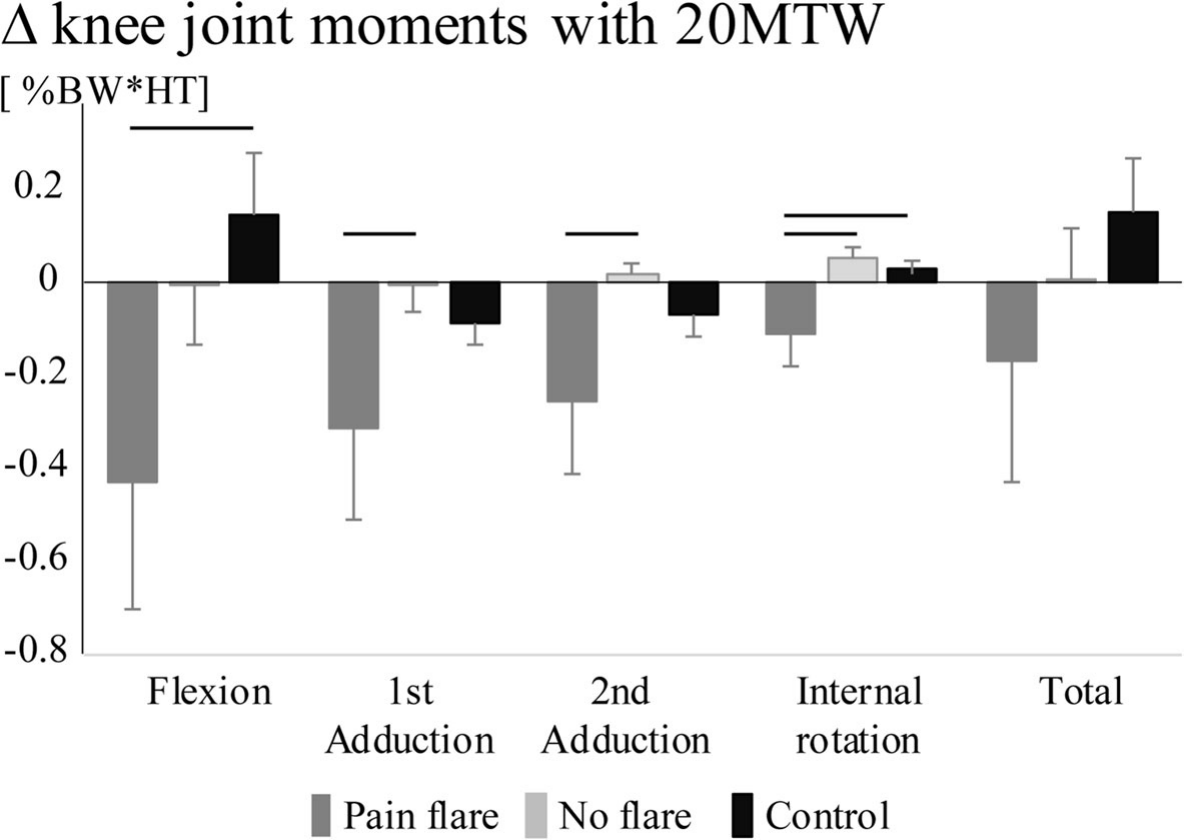
Mean changes (±SE) in knee joint moments with the 20-min treadmill walk (20MTW). Black bars indicate significant post-hoc group differences α = 0.05

In response to the 20MTW there was a main group effect for the change in 2nd peak knee adduction moment (*p* = 0.05), knee internal rotation moment (*p* = 0.005) and ankle eversion moment (*p* = 0.02) and a trend for a group effect for the knee flexion (*p* = 0.08) and 1st peak knee adduction moments (*p* = 0.1). There were no main effects for condition. There was a significant group by condition interaction effect for the hip extension moment. The response to the 20MTW did not differ between the no flare and control group (*p* > 0.1 and d < 0.4 for all comparison) but did differ between the pain flare and other groups. The pain flare group displayed a larger decrease compared to no flare group for the 1st peak knee adduction moment (*p* = 0.03, d = 0.60)*,* 2nd peak knee adduction moment (*p* = 0.02, d = 0.68), knee internal rotation moment (*p* = 0.002, d = 0.82) and ankle eversion moment (*p* = 0.02, d = 0.59) (Fig. 2). The effect size for the difference in changes for pain flare vs no flare was moderate for the knee flexion moment (*p* = 0.1; d = 0.52). In addition, there were greater decreases for the pain flare vs control group for the knee flexion moment (*p* = 0.03; d = 0.66), knee internal rotation moment (*p* = 0.005, d = 0.82) and ankle eversion moment (*p* = 0.007, d = 0.73). The decreases in the 1st and 2nd peak knee adduction moment were on average 3.5 times greater for the pain flare group as compared to the control group however the large variance between participants in the change led to moderate effect sizes (*p* = 0.1, d = 0.49; *p* = 0.9; d = 0.46). There were no differences in the magnitude of change in DCCR for either muscle grouping (Table 5).

## Discussion

The aim of this study was to quantify the impact of walking mechanics and muscle co-activation on changes in OA pain with a bout of exercise and then to examine the biomechanical response to increased pain. In response to the 20MTW almost half of the OA participants reported clinically relevant increases in pain. Those OA participants who experienced a pain flare had greater joint moments at baseline as compared to those who did not experience a pain flare and greater muscle co-activation as compared to both no flare and control groups. This suggests that individuals with OA prone to exercise-induced pain flares have greater joint loads and are more similar to healthy controls as compared to individuals with OA who do not experience pain flares. In response to the 20MTW, there were significantly greater changes in gait mechanics for the pain flare group as compared to both control and no flare OA groups. Thus despite baseline gait that may increase risk for pain, individuals who experienced a pain flare are able to rapidly adapt their mechanics to small variations in joint pain. These results also suggest that individuals who are more susceptible to these pain flares may experience the greatest benefits of biomechanical intervention such as variable stiffness shoes [33] or gait retraining [34, 35].

Beyond the observations of increased pain with activity as well as greater pain with faster as compared to slower walking [4, 22], there is limited literature on the role of in-vivo gait mechanics on pain in OA. Supporting a key role of gait mechanics in the OA pain experience, the individuals who did not experience a pain flare tended to have smaller magnitude joint moments at the knee as compared to the controls and OA participants who experienced pain flares. In addition, for those individuals who experienced a flare, the knee flexion and adduction moments, surrogate markers for the loading at the knee joint, were not different compared to controls and there was greater co-activation of the medial compared to lateral musculature despite the pain flare group walking at a slower speed than the controls. Together this suggests greater medial joint loading before the onset of pain in this flare group. The lack of a difference between OA pain flare and control groups is somewhat surprising as we might expect those with more severe symptoms in the flare group to have adapted their gait to a greater degree. However, the greater change in pain with a 20MTW for individuals with greater joint moments fits with initial evidence from studies examining load modifying shoe interventions that indicates a reduction in the external knee adduction moment can lead to moderate pain relief [15, 36]. Together these findings suggest that “poor” mechanics resulting in greater joint loading may contribute to development of pain during weight bearing activity. Those individuals with OA who have not adapted their gait to reduce loading as compared to healthy older adults may be at greater risk for pain flares but may also be at greater risk for disease progression [37].

Individuals with knee pain are hypothesized to adopt neuromuscular strategies to increase knee joint stiffness, decrease joint movement and alter movement patterns to shift or reduce tissue loads and thus limit pain [18]. The dominant response to increased pain in the OA flare group was a reduction in the magnitude of loading via a reduction and redistribution of loads through decreases in knee joint moments. For the pain flare group there were significantly greater decreases in the peak knee flexion, 1st and 2nd peak knee adduction and peak internal rotation moments as compared to the no flare or control groups with the 20MTW. The changes in peak knee flexion and first peak knee adduction moments were on the order of 12–15% for the flare group and 0–4% for the other groups. This response in the pain group is consistent with the response to experimental pain models that produce acute pain in a healthy joint using an intra-articular injection of hypertonic saline [19]. These results suggest that, even in the presence of baseline pain, the motor system in OA remains highly sensitive and able to adapt on a short time scale to small magnitude variations in OA-related joint pain that may occur over a day or a week’s time [3, 38].

For the pain flare group the magnitude of the pain change (vNRS ~ 1.5 points) was smaller than in a prior study using a similar walking protocol in patients with moderate to severe OA [4]. This may be expected as the current participants, in contrast to the previous, had less severe pain at baseline (vNRS ~ 1.89 points), reported a lesser impact of OA pain and symptoms in daily life and were not required to report an exacerbation of pain prior to enrollment. Additional support for this impact of baseline symptoms on the evoked pain flare magnitude is provided by the tendency for the participants in the OA pain flare group to have greater symptom severity as compared to the no flare group. Greater baseline pain may be indicative of greater inflammation or bony pathology and thus a greater mechano-sensitivity of the tissue [39] during walking. Participants in this study were recruited to have mild to moderate but not severe OA, as the potential to intervene and improve OA-related quality of life for a significant number of years using exercise or biomechanical interventions is the greatest in this population.

Why some OA participants may have adapted their gait to off-load the diseased joint while others have not is not clear. Understanding the factors that contribute to a beneficial gait adaptation at baseline may facilitate efforts to improve exercise adherence for symptomatic OA patients. The off-loading response to the pain increase with the treadmill walk suggests the motor system still responds in the expected way to a pain stimulus, despite the lack of adaptation at baseline in these individuals. However, a greater variance in biomechanical response in the flare group may indicate that there are some subject-specific adaptation strategies to increased OA-related pain. Of note, there were not differences in the DCCR following the 20MTW, a surprising finding given that prior experimental pain work shows a significant inhibition of the knee extensors with pain [40]. This may be due to limitations in the DCCR metric but may also be true differences in the motor system response to fluctuations in chronic pain versus the onset of acute experimental pain. As with most co-contraction metrics, the DCCR only quantifies the relative activation of the selected muscles. However there was not a change in the net activations (i.e. value of the numerator or denominator) following the 20MTW for any group.

While patient reported symptomatic severity of knee OA was captured, a key limitation of this study is a lack of documentation of the OA structural severity in the participants. Magnetic resonance imaging to document the presence and severity of cartilage thinning and pain-producing pathology such as synovitis, bone marrow lesions or meniscal damage [41, 42] may provide insight to why some individuals experience greater pain with exercise. Specifically, differences in the type or location of pain-producing pathologies such as bone marrow lesions [43] or more lateral vs medial compartment disease may impact both the magnitude of an exercise induced pain flare and the biomechanical response to increased pain. Additional studies to investigate the potential for variations in knee extensor muscle function, structural severity, bone marrow lesions or synovitis to impact the gait response are warranted. In this study, we only examined a single exercise stimulus, moderate walking. To translate these finding to the general public, further study is needed to determine the mechanical loading characteristics that have the greatest impact on exercise-induced pain and to quantify the biomechanical response for a broader range of activities of daily living such as balance, stair ascent and descent and rising from a chair.

## Conclusions

Exercise induced pain flares represent a significant barrier for individuals with OA to meet recommendations for physical activity and adhere to exercise interventions. The results of this study suggest that individuals who have adapted their gait to reduce knee joint loads may be less susceptible to exercise induced pain flares. This highlights a potential role of gait biomechanics in short term OA pain fluctuations. The study findings also suggest that despite the chronic nature of OA pain, OA gait patterns are not fixed and the motor system’s ability to respond to nociceptive stimuli in OA remains intact. The resulting changes in joint loading due to periodic fluctuations in pain level may have both beneficial or detrimental cartilage health and long-term OA outcomes and thus should not be ignored.

## Additional files

Additional file 1: **Table S1.** Baseline knee angles for Faster than preferred speed overground walking. * indicates a significant main group effect. (DOCX 68 kb)

Additional file 2: **Table S2.** Baseline external knee moments (%BW x Ht) for Faster than preferred speed overground walking. * indicates a significant group effect. (DOCX 63 kb)

## Abbreviations

20MTW: 20 min treadmill walk
DCCR: Directed co-contraction ratio
EMG: Electromyography
OA: Osteoarthritis
vNRS: Verbal numeric rating scale
