# Supplementary material for: Gait mechanics contribute to exercise induced pain flares in knee osteoarthritis
Source: BMC Musculoskelet Disord. 2019 Mar 14;20:107. doi: 10.1186/s12891-019-2493-4 (PMC6419357; doi:10.1186/s12891-019-2493-4)
Supplement: Supplementary file 1 [file 12891_2019_2493_MOESM1_ESM.docx]

Table S1: Baseline knee angles for <u>Faster than preferred</u> speed overground walking. * indicates a significant main group effect.						
	Overground speed * (m/s)	Cadence* (step/min)	Heel-strike Flexion* (deg)	Peak Flexion (deg)	Toe-off Flexion* (deg)	Average In/ext rotation* (deg)
Pain Flare (n=8)	1.70	63.75	10.69	23.49	58.35	3.04
SE	0.12	2.37	2.06	2.27	3.91	1.06
No Flare (n=11)	1.77	69.54	8.74	19.86	62.57	-0.84
SE	0.07	1.01	1.90	1.79	2.31	1.54
Controls (n=15)	1.89	69.64	4.26	22.22	67.66	-0.01
SE	0.04	1.48	1.13	1.13	0.92	1.15
