# Supplementary material for: Gait mechanics contribute to exercise induced pain flares in knee osteoarthritis
Source: BMC Musculoskelet Disord. 2019 Mar 14;20:107. doi: 10.1186/s12891-019-2493-4 (PMC6419357; doi:10.1186/s12891-019-2493-4)
Supplement: Supplementary file 2 [file 12891_2019_2493_MOESM2_ESM.docx]

| Table S2: Baseline external knee moments (%BW x Ht) for Faster than preferred speed overground walking. * indicates a significant group effect. | | | | | | |
|---|---|---|---|---|---|---|
| | 1st Peak vertical GRF BW | Flexion* | 1st Adduction | 2nd Adduction* | Internal rotation* | Total reaction* |
| Pain Flare (n=7) | 1.16 | 3.19 | -3.31 | -2.22 | -0.89 | 4.70 |
| SE | 0.03 | 0.28 | 0.42 | 0.39 | 0.10 | 0.32 |
| No Flare (n=11) | 1.14 | 4.04 | -3.08 | -1.35 | -0.70 | 5.20⁺ |
| SE | 0.04 | 0.39 | 0.35 | 0.22 | 0.07 | 0.35 |
| Controls (n=15) | 1.22 | 5.83 | -3.92 | -2.18 | -1.09 | 7.14 |
| SE | 0.02 | 0.481 | 0.43 | 0.32 | 0.10 | 0.56 |
